# Mapping and Identifying a Candidate Gene (*Bnmfs*) for Female-Male Sterility through Whole-Genome Resequencing and RNA-Seq in Rapeseed (*Brassica napus* L.)

**DOI:** 10.3389/fpls.2017.02086

**Published:** 2017-12-13

**Authors:** Changcai Teng, Dezhi Du, Lu Xiao, Qinglan Yu, Guoxia Shang, Zhigang Zhao

**Affiliations:** State Key Laboratory of Plateau Ecology and Agriculture of Qinghai University; Key Laboratory of Qinghai Province for Spring Rapeseed Genetic Improvement, Spring Rapeseed Research and Development Center of Qinghai Province, National Key Laboratory Breeding Base-Key Laboratory of Qinghai Province for Plateau Crop Germplasm Innovation and Utilization, Institute of Spring Rapeseed, Academy of Agriculture and Forestry Sciences, Qinghai University, Xining, China

**Keywords:** *Brassica napus* L., female-male sterility, mutant, whole-genome resequencing, RNA-seq

## Abstract

In oilseed crops, carpel and stamen development play vital roles in pollination and rapeseed yield, but the genetic mechanisms underlying carpel and stamen development remain unclear. Herein, a male- and female-sterile mutant was obtained in offspring of a (*Brassica napus* cv. Qingyou 14) × (Qingyou 14 × *B. rapa* landrace Dahuang) cross. Subsequently, F_2_–F_9_ populations were generated through selfing of the heterozygote plants among the progeny of each generation. The male- and female-sterility exhibited stable inheritance in successive generations and was controlled by a recessive gene. The mutant kept the same chromosome number (2*n* = 38) as *B. napus* parent but showed abnormal meiosis for male and female. One candidate gene for the sterility was identified by simple sequence repeat (SSR) and insertion deletion length polymorphism (InDel) markers in F_7_–F_9_ plants, and whole-genome resequencing with F_8_ pools and RNA sequencing with F_9_ pools. Whole-genome resequencing found three candidate intervals (35.40–35.68, 35.74–35.75, and 45.34–46.45 Mb) on chromosome C3 in *B. napus* and candidate region for *Bnmfs* was narrowed to approximately 1.11-Mb (45.34–46.45 M) by combining SSR and InDel marker analyses with whole-genome resequencing. From transcriptome profiling in 0–2 mm buds, all of the genes in the candidate interval were detected, and only two genes with significant differences (BnaC03g56670D and BnaC03g56870D) were revealed. BnaC03g56870D was a candidate gene that shared homology with the *CYP86C4* gene of *Arabidopsis thaliana*. Quantitative reverse transcription (qRT)-PCR analysis showed that *Bnmfs* primarily functioned in flower buds. Thus, sequencing and expression analyses provided evidence that BnaC03g56870D was the candidate gene for male and female sterility in the *B. napus* mutant.

## Introduction

Flowers, including the sepals, petals, stamens, and carpels, are the reproductive organs of flowering plants. The stamens are the male reproductive organs, comprising the anthers, and filament (Scott et al., 2004). The carpels, which are located in the fourth innermost whorl of the flower, are complex organs that differ widely in form between species for successful pollination, seed maturation, and seed dispersal. Most carpels of different flowering plants exhibit similar structures, including an ovary that encloses the ovules, a style, and a stigma (Fu et al., 2014).

The life cycle of flowering plants involves both sporophytes and gametophytes. Sporogenesis and gametogenesis consist of two sequential processes, corresponding to transition from the sporophytic stage to the gametophytic stage. Macrosporogenesis is the differentiation of hypodermal cells in the ovule primordium into megasporocytes, and microsporogenesis is the differentiation of hypodermal cells in the anther into microspores. The sporocytes and meiocytes subsequently undergo meiosis, with the sporocytes giving rise to microspores in male organs and the meiocytes giving rise to megaspores in female organs. Gametogenesis is the process by which haploid spores develop into mature gametophytes. Gametophyte development and successful reproduction require normal meiosis and mitosis to form anthers and ovules. Subsequently, an egg cell within the embryo sac is fertilized by a sperm cell, generating a sporophyte and completing the life cycle (Scott et al., 1991a,b, 2004; Ding et al., 2012).

With the continuous development of science and technology, next-throughput sequencing methods, such as ABI SOLiD, Illumina Solexa, and Roche 454 systems, have dramatically increased the sequencing efficiency and reduced sequencing cost making research on genomes and transcriptomes easier and more feasible (Schuster, 2008). *De novo* genome sequencing and whole-genome resequencing have been applied in many higher plants thus far, including *Arabidopsis thaliana* (The Arabidopsis Genome Initiative, 2000; Cao et al., 2011), *Brassica rapa* (Wang et al., 2011, 2016), *Brassica juncea* (Yang et al., 2016; Zhao et al., 2017), *Cucumis sativus* (Huang et al., 2009; Lu et al., 2014), *Capsicum* (Qin et al., 2014), *Oryza sativa* (Huang et al., 2011; Takagi et al., 2015), *Zea mays* (Xu et al., 2014), *Brassica oleracea* (Liu et al., 2014), and *Brassica napus* (Chalhoub et al., 2014). Additionally, the transcriptomes of many higher plants, including *A. thaliana* (Chen et al., 2010; Torti et al., 2012), *Myrica rubra* (Feng et al., 2012), *Gossypium hirsutum* (Liu et al., 2016), *B. chinensis* (Zhou et al., 2016), *Fagopyrum* (Logacheva et al., 2011), *Rosa chinensis* (Guo et al., 2017), *Lens culinaris* Medikus (Singh et al., 2017), and *B. napus*, have been sequenced for various purposes, such as fertility studies (Yan et al., 2013; An et al., 2014; Fu et al., 2014). Concerning genome *de novo* sequencing, genomes are usually repetitive, polyploidy, and heterozygous, thereby complicating genome assembly (Michael and VanBuren, 2015), which provides the reference for whole-genome resequencing and transcriptome sequencing. In whole-genome resequencing, differentially expressed genes (DEGs) are identified based on genome *de novo* sequencing, commonly using a bulked segregant analysis (BSA) strategy, in which linkage to target genes are determined in two kinds of individuals with different or even adverse phenotypes by genotyping a single pair of bulked DNA samples (Wang et al., 2016). In contrast, in RNA-Seq analysis, almost all expressed genes are obtained (particularly low-abundance genes) (An et al., 2014), leading to an exhaustive analysis of the abundant differences in the expression of various genes and pathways. RNA-Seq is also used to identify novel genes and single-nucleotide polymorphisms (SNPs) for genome-wide association studies (GWASs) (Trick et al., 2009; Harper et al., 2012).

Polyploidy is prevalent in the plant genome and may lead to extensive genetic redundancy, but it is generally absent in animals (Roulin et al., 2013). The fate of duplicated genes has been studied by whole-genome sequencing or RNA-Seq in organisms such as *Glycine max* (Roulin et al., 2013), *Triticum aestivum* (Pont et al., 2011), and *B. napus* (Yi et al., 2010). Duplicate genes of multiple copies diverge by different sequences or functions in a process called pseudogenization, subfunctionalization, or neofunctionalization (Pont et al., 2011; Roulin et al., 2013).

In crops, significant male sterility but little female sterility and even less male-female sterility is observed. At present, the male- and female-sterile crops have been reported in *Lycopersicum esculentum* (Hao et al., 2017), *G. max* (Kato and Palmer, 2003; Baumbach et al., 2016), and *Citrullus lanatus* (Zhang et al., 2012). A previous genetic analysis revealed that a male- and female-sterile mutant was controlled by a single recessive gene, designated the *B. napus* male-female sterility (*Bnmfs*) mutant. The mutant was obtained by distant hybridization between *B. napus* and *B. rapa* and exhibits normal fertility, and the plant trait was segregated in the F_2_ generation and maintained by heterozygotes. However, the narrow genetic base of *B. napus* was implied in the bottlenecks of its breeding, and the discovery of novel male- or female-sterile lines could provide breeding germplasm resources. Through the study of the mutant, the cause of male and female sterility was analyzed. If male or female fertility could be restored, the novel mutant could provide excellent material for breeding and for male and female fertility research. In the present study, whole-genome resequencing was performed on DNA from the leaves of 20 fertile and sterile plants using an Illumina high-throughput sequencing platform, and 12 SSR and InDel markers linked to the *Bnmfs* sterility gene were identified within a short interval. To further identify candidate genes, the transcriptomes of young flower buds of fertile and sterile plants with a length of 0–2 mm were sequenced. The aims of the present study were to identify a candidate gene responsible for the difference between fertile and sterile buds to characterize the associated bioprocesses and determine related gene functions. The results of this work will facilitate the cloning of *Bnmfs* and establish the foundation of rapeseed breeding for the illustration of the molecular mechanisms underlying sterility.

## Materials and methods

### Materials and population construction

**Figure 1:**
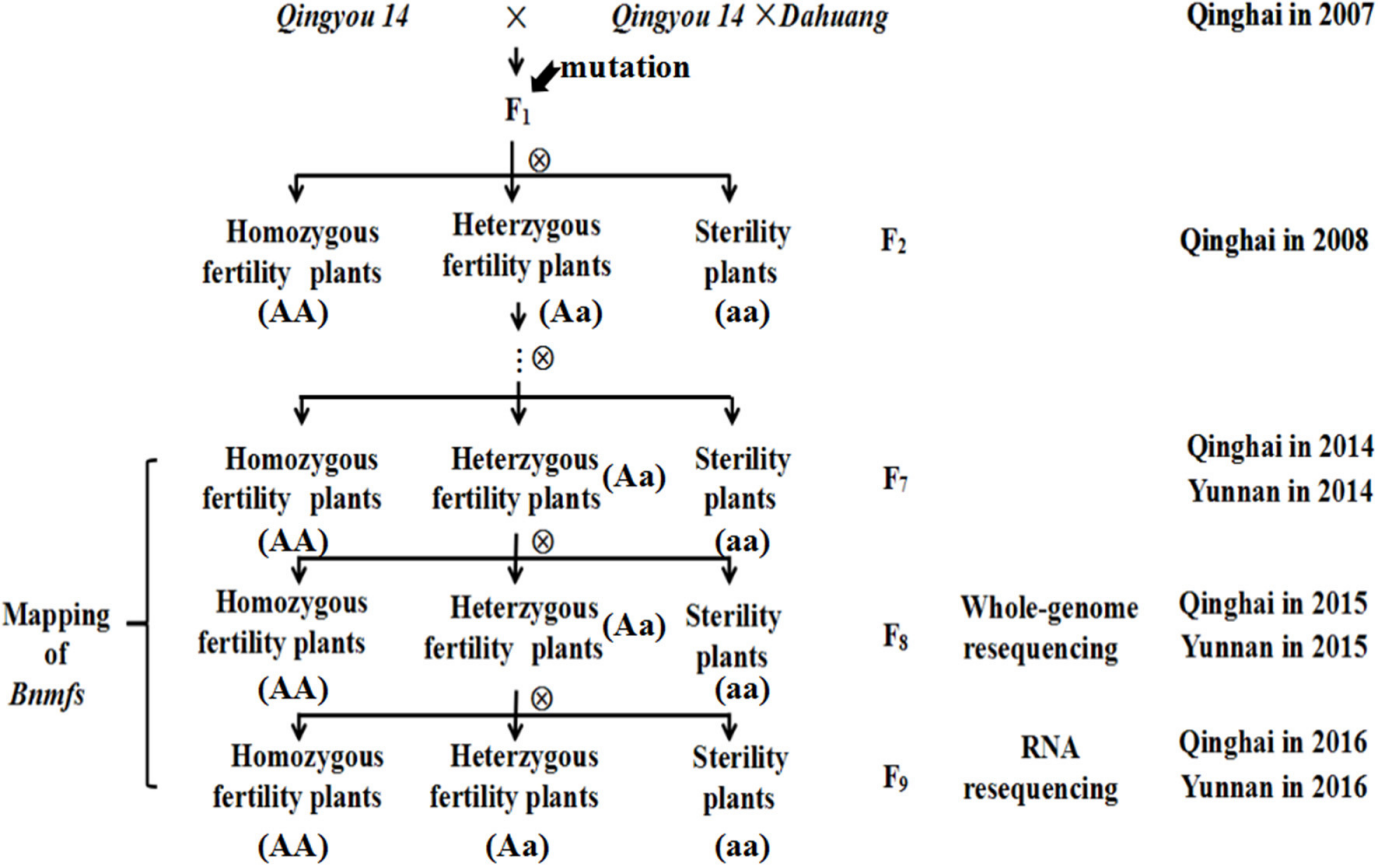
Flow diagram for the population construction of mutant.

The male- and female-sterile mutant, which was generated by interspecific crosses and exhibited stable inheritance, was obtained from a hybridization between the *B. napus* cultivar “*Qingyou 14*” as the recipient parent and a “*Qingyou 14*” × “*Dahuang*” F_1_ hybrid as the donor parent. *Dahuang* is a *B. rapa* landrace that was discovered in the Qinghai-Tibetan plateau. All F_1_ plants exhibited fertility. The heterozygotic plants were maintained in subsequent generations, and selfing generated the F_2_ and F_3_ population. F_3_ plants were harvested separately and sown to obtained the F_4_ population. Multiple selfings were performed to produce NILs for further genetic analysis and mapping. After flowering, population separation was analyzed, and the data were recorded. The materials were cultivated in an experimental field of the Academy of Agriculture and Forestry Sciences of Qinghai University from March to September in 2008-2016 and in Yuanmu, Yunnan Province from October to March in 2014-2016 (Figure 1).

### Pistil and stamen development

Pistil and stamen development was evaluated according to Han et al. (2013) with minor modifications. The buds of different developmental stages were collected based on flower bud length. The collected materials were immediately fixed in FAA solution (50% ethanol 90 ml, acetic acid 5 ml, formalin 5 ml, and glycerol 5 ml), and the air in flower buds was subsequently evacuated for 30 min and saved at room temperature or at 4°C when the test materials were stored for longer periods. To observe pistil and stamen development, the flower buds were transferred to 70% ethanol, and the sepals and petals were removed to expose the anthers and ovules. Subsequently, the samples were treated with a ethanol series (50, 30, and 10%) and transferred to distilled water. The samples were stained with 1% eosin and hematoxylin diluted solution for 96 h, followed by rinsing 3 times in distilled water and dehydration in a graded ethanol series from 30 to 100% ethanol (30% steps for 3 h each). The dehydrated samples were then cleared in graded mixtures of dimethylbenzene: anhydrous alcohol (1:4, 1:2, 2:1) and pure dimethylbenzene, with each step lasting 2 h. Finally, after infiltration and embedding in paraffin, the pistils and stamens were transversely sectioned into 8-μm slices, and bright-field images of the samples were obtained using a Nikon microscope (Nikon, Japan).

### Cytological analysis

Flower buds were collected at the appropriate sizes and placed in Carnoy solution (3:1 ethanol: glacial acetic acid, v/v) until the samples were discolored. The anther was dissected from the flower bud using forceps and subsequently softened in hydrochloric acid solution at 60°C, placed on a glass slide, mashed, stained with acetocarmine for a few seconds, covered with microscopic glass, and examined under a microscope.

The ovaries from young flower buds were collected from 9 to 11 in the morning to determine the chromosome numbers. The ovaries were first pre-treated with a 2 mM 8-hydroxyquinoline solution for 3 h at room temperature and then subsequently fixed in Carnoy solution for 24 h and stored in 70% ethanol at −20°C until further use. Cytogenetic observations were performed according to methods described by Li et al. (1995).

### Molecular mapping of the mutant gene

The F_7_-F_9_ populations, including 955 sterile individuals, were used to map the mutant gene. Total DNA was leached from young leaves using the CTAB method (Warude et al., 2003). Two fertile pools were constructed using mixed DNA from 12 randomly selected fertile plants, and two sterile pools were similarly constructed. The four pools were screened for molecular markers with 2453 PCR primer pairs [1536 AFLPs (*EcoRI/MseI, PstI/MseI, ScaI/MseI*) (Yi et al., 2006) and 917 SSRs] to identify polymorphic markers. Next, all sterile individuals in F_7_ were screened for the polymorphic markers for linkage analysis. To further narrow the scope, SSR and InDel primers were designed within the target chromosome region based on the *Brassica* Database (BRAD) (http://brassicadb.org/brad/), the *B. napus* Database (http://www.genoscope.cns.fr/brassicanapus/cgi-bin/gbrowse_syn/colza/) and whole-genome resequencing. The data were subsequently collected and transformed according to JoinMap 3.0, and a linkage map was constructed.

### Localization of *Bnmfs* by whole-genome resequencing

Whole-genome resequencing using BSA was conducted on two DNA mixed specimens from 20 fertile plants and 20 sterile plants in F_8_ generation. The libraries were produced using the Truseq Nano DNA HT Sample Preparation kit (Illumina USA) following the manufacturer's recommendations, and index codes were added to attribute sequences to each sample. The DNA sample was fragmented, PCR amplified, and purified. The products were sequenced on an Illumina HiSeq™ PE150 system. The reference genome of *B. napus* was downloaded from (http://www.genoscope.cns.fr/brassicanapus/data/Brassica_napus_v4.1.chromosomes.fa.gz). Sequencing data were subjected to quality control and mapped to the reference genome using Burrows-Wheeler Aligner software (settings: mem-t 4-k 32-M-R) (Li and Durbin, 2009). Alignment files were converted to BAM files, and potential PCR duplications were removed using SAM tools software (Li and Durbin, 2009). Variant calling was performed for all samples using the Unified Genotyper function, and SNPs were selected by using the Variant Filtration parameter in GATK (McKenna et al., 2010). The read depth information for homozygous SNPs of the two pools was used to calculate the SNP index, and the fertile pool was used as the reference. The ΔSNP index was calculated as the SNP index of the fertile pool minus that of the sterile pool. Whole-genome resequencing was performed by the Novogene Bioinformatics Technology Co., Ltd. (Beijing, China).

### RNA extraction and illumina sequencing

Total RNA was separated from 0 to 2 mm buds to obtain six mixed RNA samples. Three fertile RNA samples were prepared as replicates from the RNA extracts of 30 randomly selected fertile buds and then separately mixed using a quantity of RNA equivalent to 10 buds; the Easyspin Rapid Plant RNA Extraction kit (Dnase I; Beijing, China) was used for these assays, following the manufacturer's protocol. Three sterile RNA samples were similarly constructed. mRNA was then reverse transcribed using the PrimeScript™ RT reagent kit (Takara, Japan), and high-quality cDNA was generated for the subsequent RNA sequencing.

After the extraction of total RNA, the mRNA was enriched fragmented into short sequences and reverse transcribed into cDNA for the synthesis of second-strand cDNA. Next, the cDNA fragments were purified, the ends were repaired, poly (A)tails were added, and the fragments were ligated to adapters. Subsequently, the size of the ligation products was determined. The products were PCR amplified and sequenced on an Illumina HiSeq™ 2500 platform (Illumina Inc., CA, USA) by Gene Denovo Biotechnology Co., Ltd. (Guangzhou, China). The reads obtained from the sequencing instruments were filtered to remove adapters and low-quality reads. Subsequently, the high-quality clean reads from all six samples were merged together and mapped to the reference sequence.

### Normalization of gene expression levels and identification of DEGs

Gene expression levels were normalized using the FPKM (fragments per kilobase of transcript per million mapped reads) method. DEGs were identified by comparing the expression levels in fertile plants and sterile plants to infer transcriptional changes. After the expression level of each gene was annotated, we compared the genes and identified genes with a fold change ≥ 2 and a false discovery rate (FDR) <0.05 as significant DEGs. For convenience, DEGs exhibiting higher expression levels in fertile flower buds than in sterile flower buds were denoted as up-regulated, whereas those exhibiting the opposite relationship were denoted as down-regulated.

### Functional annotation, gene ontology (GO), and kyoto encyclopedia of genes and genomes (KEGG) classification

To determine the main biological functions of DEGs, all expressed genes were functionally annotated using the KEGG database. The reads from the fertile and sterile samples were separately mapped to the reference genome assembly using Tophat2 (Kim et al., 2013). Subsequently, the differentially expressed unigenes were obtained using Cufflinks (Trapnell et al., 2010).

Gene Ontology (GO) is an international standardized gene functional classification system that offers a dynamic, updated, controlled vocabulary, and a strictly defined concept to comprehensively describe properties of genes and their products using three ontologies: molecular function, cellular component, and biological process. KEGG is the major public pathway-related database. Pathway enrichment analysis identified significantly enriched metabolic pathways or signal transduction pathways in DEGs compared with the whole genome background.

### Quantitative real-time PCR (qRT-PCR) analysis

Total RNA was extracted from the same fertile and sterile plants for RNA-Seq, and cDNA was synthesized using the PrimeScript™ RT reagent kit (TAKARA BIO Inc., Shiga, Japan). Two significant DEGs and one random DEG were determined by molecular markers, whole-genome resequencing and RNA sequencing. Eight independent biological replicates and three technical replicates were employed for qRT-PCR validation. Primers for qRT-PCR were designed based on three gene sequences in Primer3 (Table 1). The reactions were performed with SYBR® Premix Ex Taq™ II (Tli RNaseH Plus; TAKARA BIO Inc., Shiga, Japan) on a LightCycler 480 instrument (Roche, Basel, Switzerland). Differences in gene expression were calculated using the 2^−ΔΔCt^ method. The *Actin* gene was used as an internal reference controls.

**Table 1:** Primer sequences of qRT-PCR expression analysis.

**Marker name**	**Primer sequences**	**Amplified unigenes**
Actin F	TCCCTCAGCACTTTCCAACAG	*Actin*
Actin R	ACACTCACCACCACGAACCAG	
Qpp-1 F	TGGAGACGCTTGATGTTGTTCCA	BnaC03g56670D
Qpp-1 R	CGTCAGTCAAGGCACCAAGC	
Qpp-2 F	TCCAAGACGTGCTCTTACGCT	BnaC03g56870D
Qpp-2 R	CGAATCCATGCACGACGTCAA	
Qpp-3 F	AAGCCCTAGAGGCGAGCAAG	BnaC03g57010D
Qpp-3 R	ACCAAACCGGTGTAACCGACA	

## Results

### Phenotypic survey of fertile and sterile flower buds

**Figure 2:**
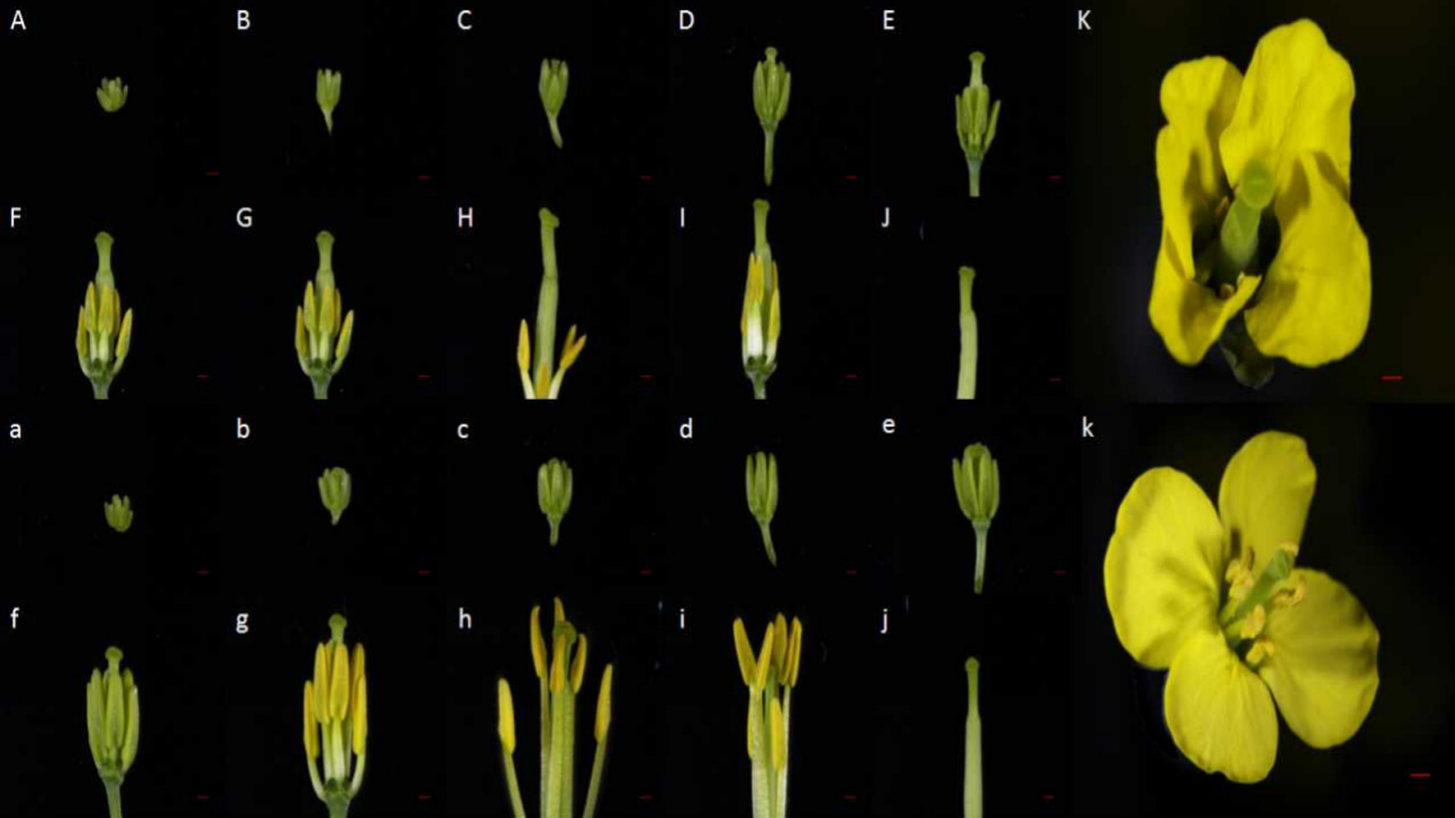
Phenotypic characterization of the sterile and fertile floral buds. **(A–K)** Phenotype of sterile flower buds. **(a–k)** Phenotype of fertile flower buds. **(A–K)** and **(a–k)** represent the sterile and fertile bud development stages, respectively. Bars = 1 mm

**Figure 3:**
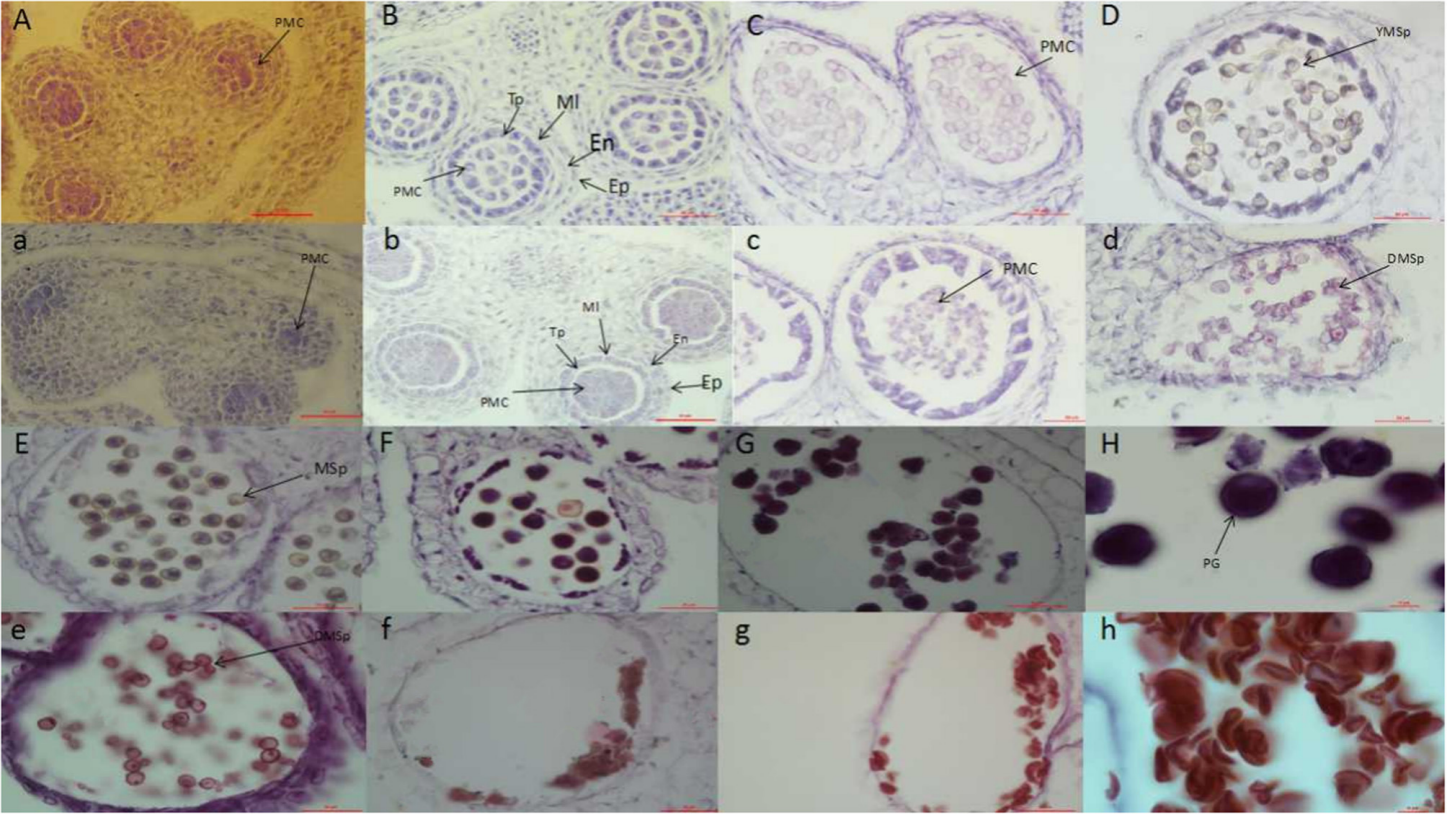
Anther development in fertile plants **(A–H)** and sterile plants **(a–h)**. PMC, pollen mother cell; Ep, Epidermal cell layer; En, endothelial cell layer; Mi, middle layer; Tp, tapetum layer; YMsp, young microspore; DMsp, degenerated microspore; Msp, microspore; PG, pollen grain. Bars = 50 μm in **(A–G)** and **(a–g)**; 10 μm in **(H)** and **(h)**.

Within the NIL population, fertile, and sterile plants exhibited a genetic ratio of 3:1 (Table 2), indicating that the mutant phenotype was controlled by a completely recessive gene. In addition, the sterile anthers did not produce pollen, as the pistils were visually normal (Figure 2). To precisely confirm the stage in which sterility occurs, the anthers and pistils of fertile and sterile plants were observed using cytology at different developmental stages. As Figure 3 indicates, normal phenotypes were observed during the early stage (Figures 3a,b). During the following stage, the volume of the tapetal cells in male-sterile plants was greater than in male-fertile plants; additionally, tetrads were not generated in the anthers (Figure 3c), and pseudo-microspores became vacuolated (Figure 3d) in the male-sterile plants. Subsequently, the tapetum degraded obviously; lipid droplets were observed around the plasmolytic pseudo-microspores, and the pseudo-microspores exhibited evident plasmolysis (Figure 3e). Furthermore, the cytoplasm of the tapetal cells disappeared; deposits of pseudo-microspore residue were observed in the nearby tapetum (Figure 3f). Few tapetal cells were present, and the anthers contained little or no residue (Figures 3g,h). By contrast, fertile anthers developed normally at all stages (Figures 3A–H).

**Table 2:** Genetic analysis of the phenotype.

**Years**	**Generation**	**Phenotype**	**NILs**			**Expected segregation**	**χ^2^ value**
			**No. of fertile individuals**	**No. of sterile individuals**	**Total individuals**		
2008	F_1_	Normal					
2014	F_7_		457	157	614	3:1	0.2557
2015	F_8_		11,42	357	1,499	3:1	1.0587
2016	Zhongshuang 11 × *BnMFS*(F_1_)	Normal	35	0	35		
2016	*BnMFS*×Zhongshuang 11(F_1_)	Normal	29	0	29		
2017	Zhongshuang 11 × *BnMFS*(F_2_)		226	65	291	3:1	0.9633
2017	*BnMFS*×Zhongshuang 11(F_2_)		27	9	36	3:1	0.0046

*χ^2^0.05, 1 = 3.84*.

To confirm the pistil defect of sterility, transverse sections of pistils were further verified. The transverse processes of the pistils were divided into six stages based on sizes ranging from 0.5 to 5 mm. In *B. napus*, after the ovule primordia arose from the inner ovary wall, a single archesporial cell functioned as the megaspore mother cell, which was in contact with the nuclear epidermis (Figures S1A,a) (Supporting information). The diploid megaspore mother cell subsequently went through meiosis and formed tetrads of haploid megaspores (Figures S1B,b), and at that stage, the outer and inner integuments began to form. Shortly after meiosis in the fertile flower buds, three megaspores degenerated, and the surviving megaspore was the functional megaspore. The outer and inner integuments enlarged until enclosing the nucleus (Figure S1C). Subsequently, the surviving megaspore went through one round of mitosis, producing a bi-nucleated cell, in which the two nuclei were parted by a large central vacuole. In addition, the female gametophyte included a small vacuole at the chalazal pole (Figure S1D). The developing female gametophyte went through second and third rounds of mitosis. A large vacuole separated the two sets of nuclei. Outer and inner integuments enclosed the nuclei, forming an embryo sac with eight nuclei (Figures S1E–H). However, in the sterile plants, megasporogenesis was normal (Figures S1a,b), but abnormalities were observed before and after the one-nucleus stage; and meiosis was not observed. Degradation subsequently occurred. Most of the female gametophytes could not undergo the one-nucleus, two-nucleus, or four-nucleus transitions to mature into embryo sacs, resulting in significant degeneration of embryo sacs at the mature stage (Figures S1c–h).

### Mitotic and meiotic chromosomes in fertile and sterile flower buds

**Figure 4:**
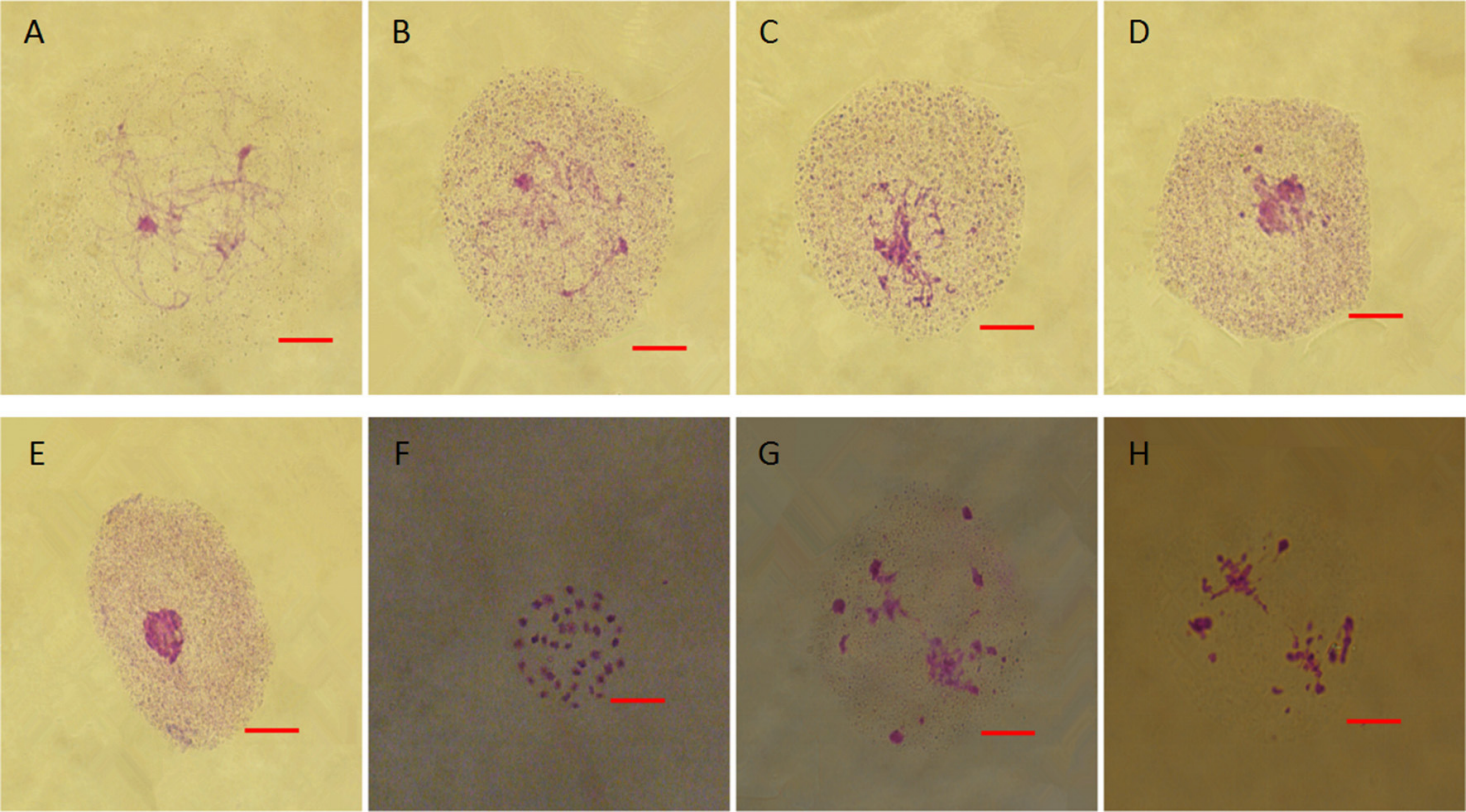
Meiosis in the anthers of sterile floral buds. **(A–D)** Similar chromatin condensation. **(E)** Darkly stained pseudo-nuclear condensation. **(F)** Few cells with coagulate pseudo-chromosomes. **(G)** Abnormal chromosome behavior. Bars = 10 μm.

From chromosome number counts in ovaries, both fertile and sterile young flower buds of 10 independent biological replicates maintained 38 chromosomes (Figures S2A,a). In fertile plants, microspore mother cells went through meiosis and generated tetrads. The images provided in Figures S3A–L illustrated the meiosis of fertile flower buds. However, in sterile flower buds, microspore mother cells gradually degraded and did not undergo meiosis (Figures 4A–E). During degradation, cells exhibited coagulation of pseudo-chromosomes (Figure 4F) and went through abnormal meiosis (Figures 4G,H).

### Genetic analysis of fertile and sterile plants

During early flowering, the F_7_–F_9_ generations were investigated for bud fertility. F_1_ plants exhibited fertile phenotypes. In the F_7_-F_9_ populations, the separation of fertile and sterile plants fit a ratio of 3:1 ($\chi^{2}<\chi_{0.05,1}^{2}$ = 3.84) (Table 2), illustrating that the mutant trait was recessive and indicating that the sterility was controlled by a completely recessive gene, designated *Bnmfs* mutant.

### Identification of three candidate intervals via whole-genome resequencing analysis

**Figure 5:**
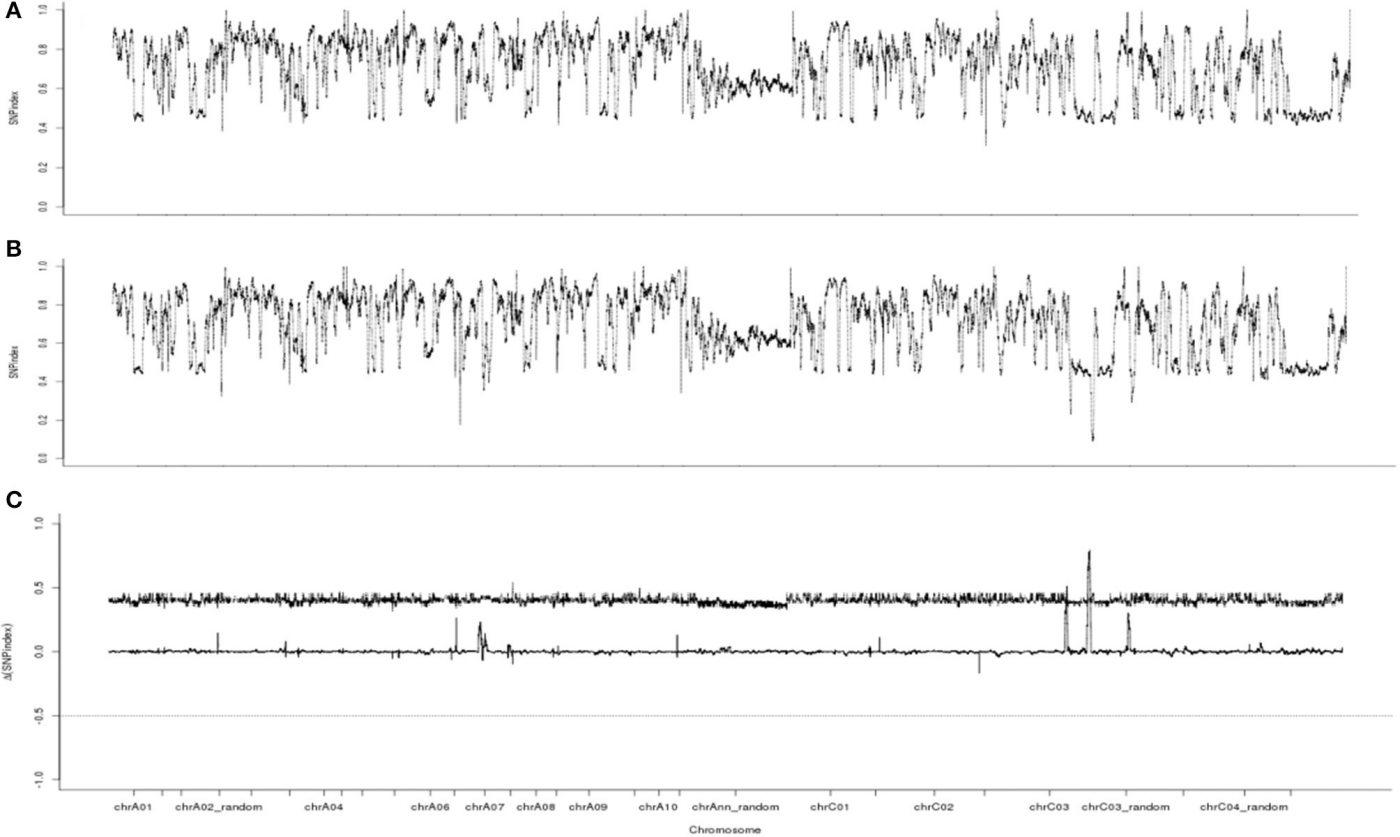
SNP index and ΔSNP index Manhattan plot graphs. **(A)** SNP index Manhattan plot graphs of the fertile pool from F_8_. **(B)** SNP index Manhattan plot graphs of the sterile pool from F_8_. **(C)** ΔSNP index Manhattan plot graphs. The line indicates the threshold value.

Whole-genome resequencing analysis using BSA could be used to identify candidate intervals, as shown in *C. sativus* (Lu et al., 2014), *B. rapa* (Wang et al., 2016), *B. juncea* (Zhao et al., 2017), and *B. napus* (Yao et al., 2017). Therefore, whole-genome resequencing analysis was conducted for our candidate gene. A total of 47.25 G of raw data was filtered to 46.80 G of clean data. The quantity and quality (Q20 ≥ 93.70% and Q30 ≥ 86.73%) of the data were analyzed. The GC content ranged from 37.90 to 38.40%. The average read depth ranged from 24.99 × to 26.20 × for the two samples, and the 95% confidence intervals of the SNP index for each read depth were obtained (Takagi et al., 2013). The clean data were aligned to the reference genome. Approximately 2,813,972 SNPs were identified between the fertile and sterile pools. The ΔSNP index was calculated, and a chart was plotted from SNP index of the two sample pools (Figure 5). Three different intervals for the candidate gene (35.40–35.68, 35.74–35.75, and 45.34–46.45 Mb) exceeding the threshold value were identified on chromosome C3.

### Mapping candidate genes to a 1.11-Mb interval based on molecular markers

**Figure 6:**
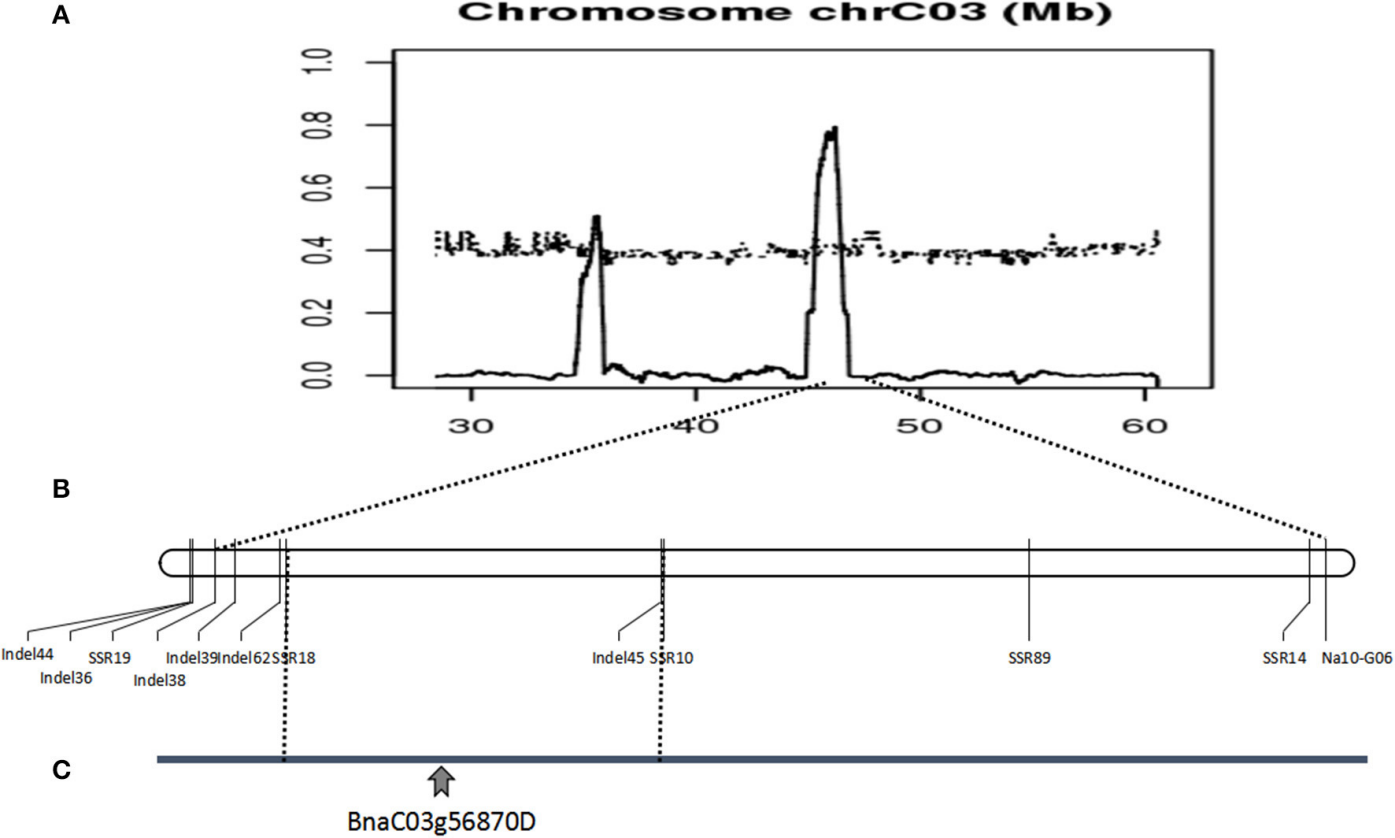
Identification and validation of the BnaC03g56870D gene on *Brassica napus* chromosome C03. **(A)** ΔSNP index graph based on the analysis of fertile and sterile plants identified a candidate gene in interval of 45.34–46.45 Mb on chromosome C03. **(B)** Linkage analysis using molecular markers confirmed the location of the candidate gene. **(C)** The location of the BnaC03g56870D by molecular markers, whole-genome sequencing and RNA-Seq.

Polymorphic screening was performed using 2453 paired PCR primers evenly distributed across the A and C genomes. Only one SSR marker (Na10-G06) and six AFLP markers exhibited polymorphisms between the mixed pools and the corresponding plants. The AFLP markers failed to transform SCAR markers. The sequence of an SSR marker located on chromosome C3 was sequenced. According to the *B. napus* genome sequence database, seventy SSR markers were developed based on an SSR marker, Na10-G06, 2M range. Four markers (Table S1) designated SSR10, SSR14, SSR18, and SSR19 were polymorphic in 157 sterile plants in the F_7_ populations Figure S4. Linkage analysis revealed that these markers included several recombinants, and were closely linked. Thus, we considered a comprehensive strategy. The three candidate intervals and the SNPs were determined through whole-genome resequencing. Sixty-four InDel markers then were designed based on the analysis of whole-genome resequencing of three different intervals on chromosome C3. The results revealed that six markers (Table S1), designated as InDel36, InDel38, InDel39, InDel44, InDel 45, and InDel62, were polymorphic in 514 sterile plants in the F_7_ and F_8_ populations. Linkage analysis demonstrated that these markers included dozens of recombinants, and fell into one interval from 45.34 to 46.45 Mb on chromosome C3. In 438 sterile plants in the F_8_ populations, 12 markers were verified. The results revealed that 12 SSR and InDel markers were polymorphic and closely linked to the candidate gene, mapped to the *B. napus* genome database, but the reason for so many recombinants was poorly understood. These results suggest that the candidate genes were located in a 1.11-Mb region of chromosome C3, from 45.34 to 46.45 Mb (Figure 6).

### Identification of expressed genes in a single candidate interval by transcriptome sequencing

To better understand the mutant gene underlying female and male sterility in flower buds, observations of the morphology were performed in different stages. During pistil and stamen development and the anther meiosis phase, the development of ~2-mm flower buds deviated in fertile and sterile plants. Thus, flower buds of <2 mm were collected from fertile and sterile plants, and the changes in gene expression responsible for different developmental patterns were analyzed. A total of 54,225,744–74,085,170 raw 150-bp paired-end reads were obtained from three fertile flower bud samples and three sterile flower bud samples by RNA sequencing. After the raw data were trimmed, an average of 65,845,755 (99.62%) and 60,635,050 (99.61%) clean reads remained from the fertile and sterile flower bud samples, respectively (Table S2).

After mapping to the reference genome, a total of 91,391 unigenes were identified and transcribed, and 72,309 and 71,776 genes were identified in fertile flower buds and sterile flower buds, respectively. One thousand and sixty-six unigenes exhibited differential expressions between fertile and sterile plants (including both up-regulated and down-regulated genes) based on a false discovery rate (FDR) ≤ 0.05 and a |log_2_FC|≥1.

Within the 1.11-Mb interval (45.34–46.45 Mb), a total of 114 unigenes were identified; 94 of the unigenes were expressed (Table S3), and two (BnaC03g56870D and BnaC03g56670D) exhibited differential expression.

### Verification of DEGs using qRT-PCR

**Figure 7:**
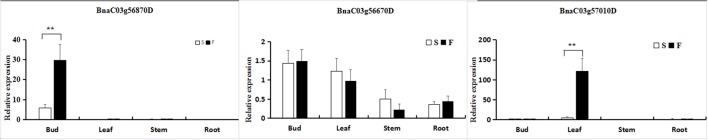
Quantitative real-time polymerase chain reaction (qRT-PCR) to confirm differential expression in various organs between fertile (F) and sterile (S) plants. The relative expression levels of three genes identified through RNA-Seq analysis and whole-genome resequencing are shown. The gene expression analysis was performed based on eight biological (buds) replicates, three biological (leaves, stems, and roots) replicates and three technical replicates. ^**^Significantly different at the 0.01 level using a *t*-test.

To confirm that the genes identified within the 45.34–46.45-Mb interval from RNA sequencing and whole-genome resequencing were differentially expressed, two significant DEGs and one random DEG (BnaC03g57010D) exhibiting differential expression patterns were selected for qRT-PCR analysis in young flower buds. The results revealed that three genes exhibited a consistent expression tendency to those obtained from the RNA-Seq data (Figure 7) despite some quantitative differences, sustaining the reliability of the transcriptome analysis.

In addition, the three differentially expressed unigenes were investigated in other organs, including the leaves, stems and roots. For the BnaC03g56870D gene Data Sheet 1, the observed expression differences were slight, except in the buds. Moreover, the expression differences of BnaC03g56670D and BnaC03g57010D were also small, except for the expression of BnaC03g57010D in the leaves.

## Discussion

The understanding of reproductive development in *B. napus* is limited, and the discovery of other mutants would add to current information on regulatory mechanisms underlying the processes. Many mutants associated with floral organ identity have been obtained, such as *dg1, fsm, ms1-9, msMOS, msp, st8, mfs1*, and *MFS* (Kato and Palmer, 2003; Ding et al., 2012; Zhang et al., 2012; Yang et al., 2014; Baumbach et al., 2016; Huang et al., 2017; Yu et al., 2017). The *dg1 O. sativa* mutant displays dwarfism, exhibiting small, rolled leaves and defected spikelets (Yu et al., 2017). The *fsm* mutant of Chinese cabbage exhibits a female-sterile phenotype (Huang et al., 2017). Yang et al. identified 11 female-fertile, male-sterile mutants (*ms1, ms2, ms3, ms4, ms5, ms6, ms7, ms8, ms9, msMOS*, and *msp*) (Yang et al., 2014). For the *st8* male- and female-sterile mutant of *G. max*, the causal genes were identified and mapped (Kato and Palmer, 2003; Baumbach et al., 2016). The *mfs1* mutant was obtained from a T2 transgenic line of *O. sativa* (Ding et al., 2012). *MFS* was identified as a male- and female-sterile progeny of an F_1_-hybrid cultivar of *C. lanatus* (Zhang et al., 2012). These genes played vital roles in the regulation of reproductive development. In the present study, *Bnmfs* was identified as a male- and female-sterile mutant with stable inheritance.

In the past few decades, molecular markers have played a vital role in target gene mapping and molecular-assisted breeding, but differentially segregating populations might exhibit different location effects in some cases, Furthermore, some populations failed to be mapped, reflecting noise from individuals. In our study, the NILs failed to map to the interval due to excessive recombination. In recent years, next-generation technology and large-scale data analyses have been applied to this area of research. Genome and RNA sequencing have enabled genotyping, providing a faster and more effective method for exploring target regions and DEGs with plenty of SNPs (Yang et al., 2012; He et al., 2014), and combined multi-omics analyses are becoming increasingly common. For example, allotetraploid cotton was analyzed with the aim of fiber improvement using *de novo* sequencing and RNA profiling (Zhang et al., 2015), and the boron efficiency of *B. napus* was revealed through whole-genome resequencing and digital gene expression (DGE) profiling (Hua et al., 2016). In the present study, a comprehensive strategy, including cytological observation, genetic study, molecular markers, whole-genome resequencing, RNA-Seq, and qRT-PCR, was employed to characterize the mutant. Ultimately, BnaC03g56870D was identified as a candidate gene for male and female sterility in *B. napus*.

In our research, morphological observations revealed that the abnormal pollen and ovules of *Bnmfs* plants influenced normal fertilization, leading to male and female sterility. Therefore, to confirm the identified candidate gene, three intervals were located using whole-genome resequencing combined with the BSA method. Molecular marker analysis excluded two of the intervals, thus narrowing the potential location of the candidate genes to a single interval. We analyzed the intervals for all genes via RNA-Seq, and two genes exhibited differential expression levels. According to the annotation of two genes in the NCBI, TAIR and the Genoscope databases (Table S4), one gene, BnaC03g56670D, related to an RNA helicase, had nothing to do with fertility. The another gene, BnaC03g56870D, cytochrome P450, family 86, subfamily C, was related to *CYP86C4* in *A. thaliana*, which was the male fertility gene in Chinese cabbage (Cao et al., 2006). Meanwhile, qRT-PCR verification indicated that Bnac03g56670D was expressed at low levels in all tissues and that there was no difference between fertility and sterility, and Bnac03g56870D was highly expressed in young flower buds but was not expressed or was expressed at low levels elsewhere.

Bnac03g56870D was mapped by molecular markers, whole-genome resequencing, RNA-Seq and qRT-PCR and was homologous to AT1G13150 of *A. thaliana*. AT1G13150 was annotated by TAIR to demonstrate that the *CYP86* family members, belonging to the cytochrome P450 family, mediated hydroxylation (Kandel et al., 2006). The *CYP86MF* gene was only expressed in floral buds and not in the leaves and stems of male-sterile plants (Cao et al., 2006). Plant P450s encoded monooxygenase. Essential P450 functions, including sterol, phenolic, alkaloid, hormone, and oxygenated fatty acid synthesis, were conserved among plant species (Cao et al., 2006). In this study, BnC03g56870D was found to be significantly expressed in the young flower buds in the fertile plants, expressed at low levels in the sterile plants, and inhibited in the leaves, stems and roots of the fertility and sterile plants (Figure 7).

In summary, the better understanding of the molecular mechanisms of fertility, based on cloning and functional analysis of the sterility gene in *B. napus*, may provide a new opportunity to enhance the heterosis utilization of oilseed rape.

## Availability of supporting data

The whole-genome resequencing and transcriptome sequencing data were submitted to NCBI BioProject with BioProject ID: PRJNA401257 (http://www.ncbi.nlm.nih.gov/bioproject/?term=PRJNA401257).

## Author contributions

Experimental design: CT and DD; Experiments: CT; Data analysis: CT and ZZ; Manuscript preparation: CT; Manuscript modification: CT, LX, GS, and QY.

### Conflict of interest statement

The authors declare that the research was conducted in the absence of any commercial or financial relationships that could be construed as a potential conflict of interest.
