# Supplementary material for: Mapping and Identifying a Candidate Gene (Bnmfs) for Female-Male Sterility through Whole-Genome Resequencing and RNA-Seq in Rapeseed (Brassica napus L.)
Source: Front Plant Sci. 2017 Dec 13;8:2086. doi: 10.3389/fpls.2017.02086 (PMC5733364; doi:10.3389/fpls.2017.02086)
Supplement: Supplementary file 5 [file DataSheet1.DOCX]

Gene sequence

BnaC03g56870D

ATGTCTTTAACAGAGCGAGTCTTTAATCATCTTTCTCTCTTCGATATATCTCTTGCGTTGTTGGGGCTGTTTGTTTTCTGTTGTTTGCGTGAGAAGTTAACCAACAAGCACGGGCCAATGCTGTGGCCAGTGTTCGGCATTACCCTAGAGTTTTTCTTTCATAAAAATGATGTCTATGGATGGGTCACAAGGTCTTTAAAAAAATCCCGAAACACGTTTCTTTACCGTGGGTTCTGGCTTGATGGATCTCATGGAGCCGTGACTTGTTCTCCTGCCAATGTTGAGTACATGCTCAAGACCAACTTCAAGAACTTCCCCAAAGGTACCTTCTTTAAAGACCGGTTTAAAGATCTCCTCGAGGATGGTATTTTTAACGCTGATGATGAGTCCTGGAGAGAGCAACGACGGGTCATCATAACCGAAATGCATTCAACTCGGTTCATGGAGCATTCCTTTCAGACAACACAACGTTTAGTAAGGAAGAAGCTGTTGAAGGTTATGGAGAGTTTCGCTAGGTCACAGGAAGCTTTTGATCTCCAAGACGTGCTCTTACGCTTGACGTTTGACATCATCTGCATCGCGGGTCTTGGAGCTGACCCGGAGACTCTAGCTCAAGATCTTCCTCAAGTTCCATTTGCTAAAGCTTTCGATGAAGCAACGGAGTCTACGTTGTTTAGGTTGATGATCCCTCCGTTTATATGGAAACCAATGAAGTTCCTTGATATAGGGTATGAGAAAGGTCTCAGGAAAGCTATTGACGTCGTGCATGGATTCGTGAACAAGATGATTATGGATCGTATCTGCATGGTCAATGATGAAGAGACGTTAGATAATAGATCAGATGTCCTTACAAGGATTATTCAAATAGAGAATAATAAAAAGGGTAACAAGATTGGACCTTCAACTATTAGGTTTTTTAGACAGTTTTGCACAAGTTTCATTTTAGCTGGACGTGACACAAGTTCTGTTGCGATTTCATGGTTCTTCTGGGTGATACAAAGACACCCACAAGTTGAAAACAAAATCATCCAGGAGATCAGACAAATCGTGAAACAGAGAGGAGATCCTTCAGACAGTAGTCTCTTCACGGTCAAGGAACTAAACAACATGGTATATCTACAAGCAGCAATTTCAGAAACTCTAAGACTTTACCCACCAATCCCTATGGAGATGAAACAAGCCATTGAAGATGATATGTTTCCAGATGGGACGTTTATAAAAAAGGGTTCAAGGGTTTACTTCTCTATCTATGCCATGGGAAGGATGGAAACAATCTGGGGTAAAGACTGTGAAGAGTTCAGACCAGAGAGATGGATCCAAGCAGGGAAGTTTGTTAGTGACGACCAATACAAATATGTTGTGTTCAATGCTGGGCCTAGGCTGTGTCTAGGGAAAACATTTGCTTACTTGCAAATGAAGATGATAGCTGCTTCAGTCTTGTTAAGGTATTCAGTCAAGGTTGCTCAAGATCATGTGGTTGTCCCGAGAGTTACTACTAACTTGTACATGAAGTACGGTCTCAAGGTGACCATCACGCCAAGGTTACAAGAAGAGACGAAACTAGAGTCATGTTCCATGTAG
