# Supplementary material for: Mapping and Identifying a Candidate Gene (Bnmfs) for Female-Male Sterility through Whole-Genome Resequencing and RNA-Seq in Rapeseed (Brassica napus L.)
Source: Front Plant Sci. 2017 Dec 13;8:2086. doi: 10.3389/fpls.2017.02086 (PMC5733364; doi:10.3389/fpls.2017.02086)
Supplement: Supplementary file 6 [file DataSheet2.DOCX]

Supplementary Material

Mapping and Identifying a Candidate Gene (*Bnmfs*) for Female-Male Sterility through Whole-genome Resequencing and RNA-Seq in Rapeseed (*Brassica napus* L.)

Changcai Teng†, Dezhi Du†, Lu Xiao, Qinglan Yu, Guoxia Shang, Zhigang Zhao*

* **Correspondence:**Zhigang Zhao:[13897474887@126.com](mailto:13897474887@126.com) †Changcai Teng and Dezhi Du contributed equally to this work.

FIGURE **S1. Ovule development in sterile and fertile plants.**

*MMC,* megaspore mother cell; *FM,* functional megaspore; *CN,* chalazal nuclei; *MN,* micropylar nuclei; *PN*, polar nuclei.

Bars = 50 μm in A-E, G and a-e, g; 10 μm in F, H and f, h.

**FIGURE S2. Chromosome number in sterile and fertile plants.**

A, Chromosome number in sterile plants; a, Chromosome number in fertile plants.

Bars = 10 μm.

**FIGURE S3. Meiosis in the anthers of fertile plants.**

A, Leptotene; B, Zygotene; C, Pachytene; D, Diplotene; E, Diakinesis; F, MetaphaseⅠ; G, AnaphaseⅠ; H, Telophase Ⅰ; I, Prophase Ⅱ; J, Metapase Ⅱ; K, Anaphase Ⅱ; L, Telophase Ⅱ.

Bars = 10 μm.

**FIGURE S4. Amplification results of SSR 19 for partial individuals in the F8 population.**

The F_8_ individuals represented homozygous fertility (*BnMFSBnMFS*), heterozygous fertility (*BnMFSBnmfs*) and sterility (*BnmfsBnmfs*).

**TABLE S1. Primer sequences of SSR and InDel markers linked to *Bnmfs*.**

**TABLE S2. Sequencing and assembly statistics for the transcriptome data of the six fertile flower buds and sterile flower buds.**

**TABLE S3. Expression of differentially expressed unigenes.**

**TABLE S4. Gene annotations of 114 genes on C3 (from 45.34 Mb to 46.45 Mb).**

**DOCX S1. BnaC03g56870D sequence**
