# Supplementary figures and images for: Mapping and Identifying a Candidate Gene (Bnmfs) for Female-Male Sterility through Whole-Genome Resequencing and RNA-Seq in Rapeseed (Brassica napus L.)
Source: Front Plant Sci. 2017 Dec 13;8:2086. doi: 10.3389/fpls.2017.02086 (PMC5733364; doi:10.3389/fpls.2017.02086)

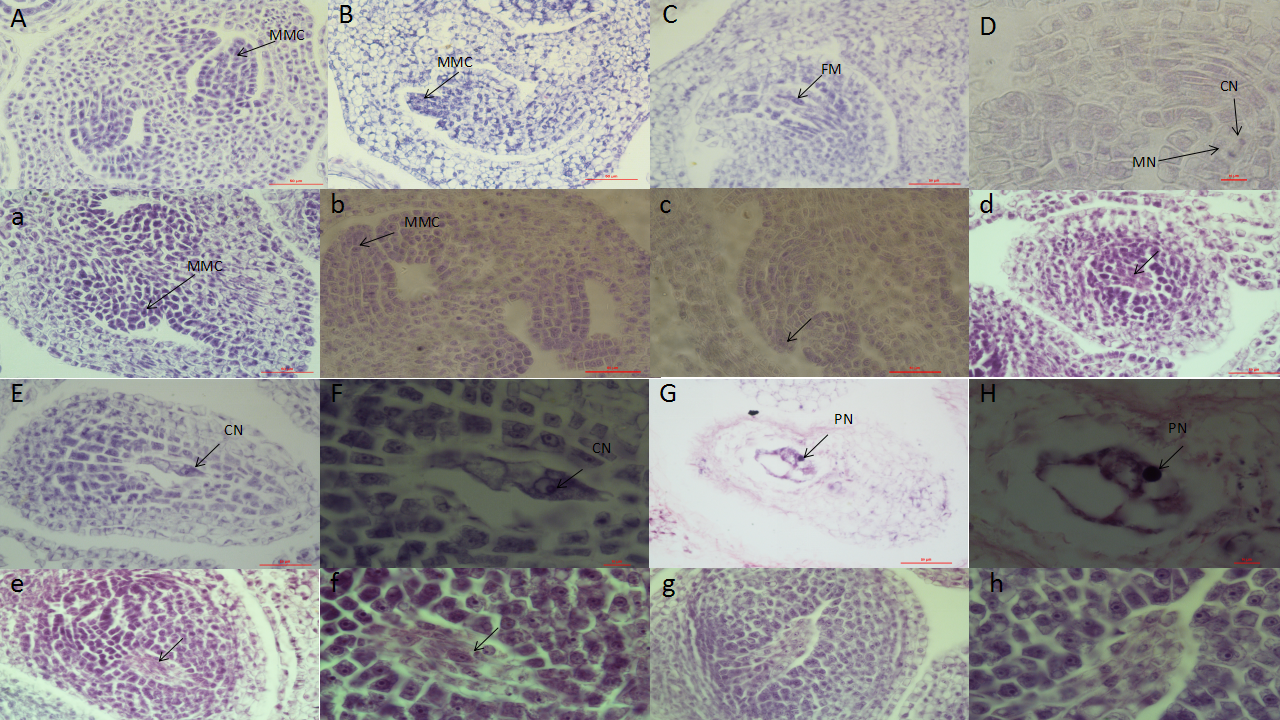

Supplement: Supplementary file 7 [file Image1.TIF]

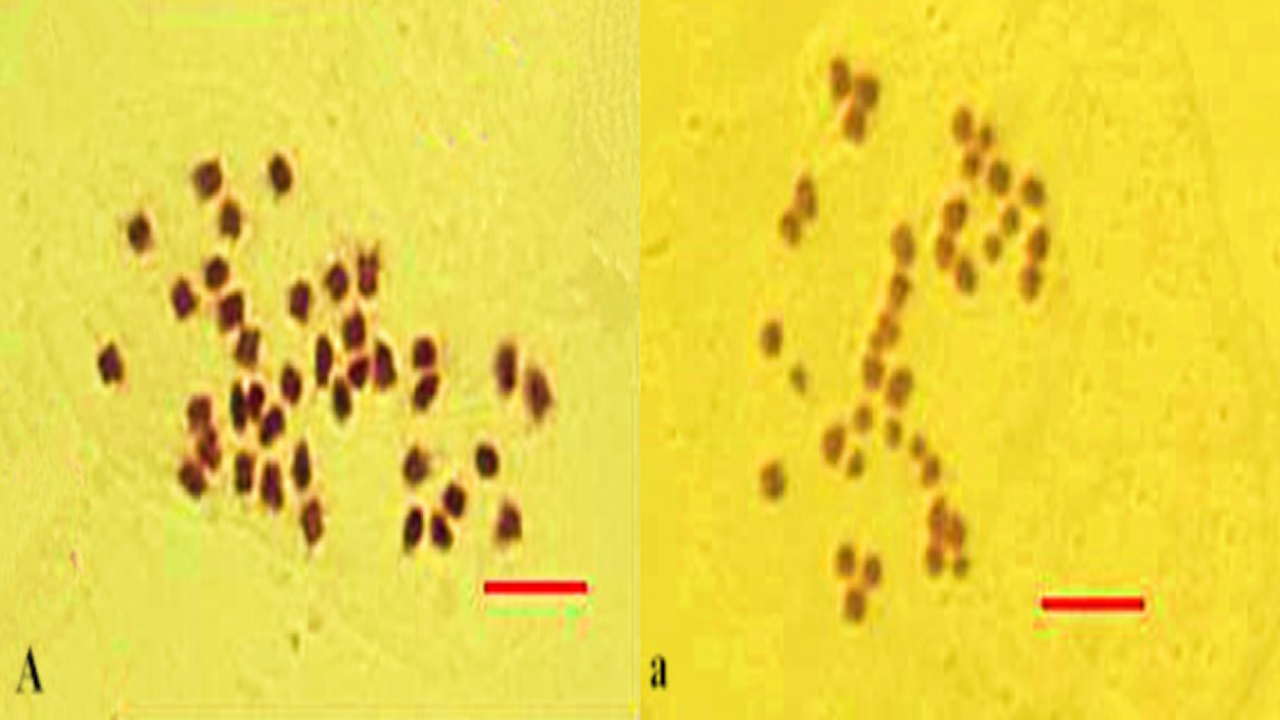

Supplement: Supplementary file 8 [file Image2.TIF]

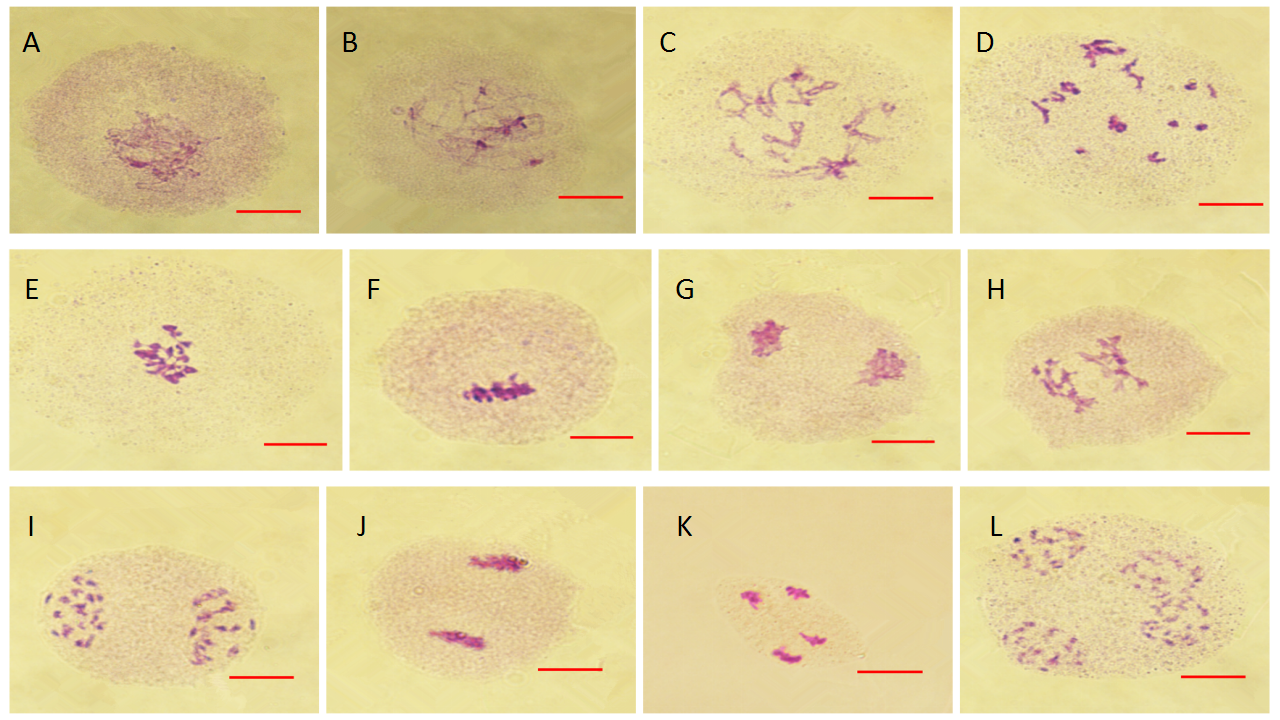

Supplement: Supplementary file 9 [file Image3.TIF]

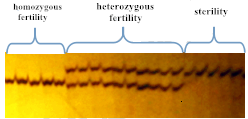

Supplement: Supplementary file 10 [file Image4.PNG]
